# Acoustic and Ultrasonic Energy Devices for Soft Tissue Dissection in General Surgery: Current Evidence and Future Directions

**DOI:** 10.7759/cureus.116963

**Published:** 2026-09-26

**Authors:** Carlos Alejandro Arragan Lezama, Luis Fernando Tandayamo Sisalima, Álvaro de Jesús Posada Manjarrez, José Luis de Jesús Arguello Hernández, Catalina Garzón Libreros, Gustavo Adolfo Sanic Coj

**Affiliations:** 1 Outpatient Care, Universidad Anáhuac Xalapa, Xalapa, MEX; 2 Primary Health Care, Universidad Central del Ecuador, Quito, ECU; 3 General Surgery, Universidad de Santander, Valledupar, COL; 4 Primary Health Care, Universidad Anáhuac Xalapa, Xalapa, MEX; 5 Primary Health Care, Universidad Icesi, Cali, COL; 6 General Surgery, Universidad San Carlos de Guatemala, Guatemala City, GTM

**Keywords:** energy-based devices, general surgery, harmonic scalpel, hemostasis, hifu, laparoscopic surgery, ligasure, soft tissue dissection, thunderbeat, ultrasonic energy

## Abstract

Acoustic and ultrasonic energy instruments have paved the way for revolutionizing soft tissue dissection, leading to the development of energy instruments in general surgery. These devices, such as the Harmonic® ultrasonic surgical system (Ethicon, Johnson & Johnson), Sonicision (Covidien/Medtronic), Thunderbeat® (Olympus Corporation), and high-intensity focused ultrasound (HIFU) devices, are high-frequency mechanical vibrating tools that can cut and coagulate tissue simultaneously with minimal thermal spread compared to traditional electrosurgery. This literature review aims to present evidence (2020-2026) published in PubMed, including mechanisms of action, clinical effectiveness, safety, comparisons of systems with varying devices, and new and potential applications of ultrasonic and acoustic energy in general surgery (both open and minimally invasive). Systematic reviews, meta-analyses, and clinical trials have demonstrated a step-by-step buildup of evidence indicating that ultrasonic devices lead to fewer inflammatory tissue responses, less blood loss, a shorter postoperative hospital length of stay, and faster surgery times than traditional electrocautery. However, there are also clinical concerns, such as the possibility of overheating the blades, the risk of thermal residues, and the cost of the devices. Future trends involve incorporating artificial intelligence (AI) and smart energy delivery into the platform, adding AI to an ultrasonic (robotic-compatible) platform with wristed articulation, and expanding the clinical uses of AI in non-invasive tissue ablation.

## Introduction and background

Soft tissue dissection is a fundamental surgical skill in general surgery, and substantial advances in the instruments used to achieve tissue division and hemostasis have occurred over the past three decades [1]. Although the evolution of surgical energy technology has progressed from conventional scalpel techniques to monopolar and bipolar electrosurgery and subsequently to advanced energy platforms, the fundamental objectives remain consistent: precise tissue dissection, effective hemostasis, preservation of surrounding tissues, and operative efficiency [2]. The increasing use of minimally invasive surgery has further increased the importance of reliable energy devices that can simultaneously dissect, coagulate, and seal tissue while limiting collateral thermal injury. In the early 1990s, ultrasonically activated surgical devices represented an important technological development, using high-frequency mechanical vibrations to produce tissue effects without directly passing electrical current through the patient [3,4].

Ultrasonic energy devices convert electrical energy into high-frequency mechanical vibrations through a transducer. These vibrations are transmitted to a titanium blade or jaw, producing tissue effects through mechanical disruption, friction, protein denaturation, and coagulation. Reported operating temperatures are generally lower than those associated with conventional electrosurgical energy, although the temperature achieved depends on the device, activation duration, tissue characteristics, and operative conditions [5,6]. This distinction may be clinically relevant when energy devices are used near vulnerable structures such as nerves, the biliary tree, ureters, and vascular pedicles, where unintended thermal spread may result in clinically important tissue injury [7,8].

Contemporary surgical energy platforms now include several technologies with different mechanisms of action, including ultrasonic devices, advanced bipolar systems, hybrid ultrasonic-bipolar devices, and emerging non-contact energy technologies such as high-intensity focused ultrasound (HIFU) and histotripsy. These technologies are used according to the surgical procedure, tissue characteristics, device availability, and surgeon preference rather than according to a single universally accepted gold-standard energy source. Consequently, comparisons between devices should consider procedure-specific efficacy, hemostasis, operative time, blood loss, thermal spread, complications, and other clinically relevant outcomes rather than assuming overall superiority of one platform [5,6].

A substantial body of comparative clinical trials, systematic reviews, and network meta-analyses has been published in recent years, providing an opportunity to reassess the current evidence regarding surgical energy technologies [9]. However, the available evidence remains heterogeneous with respect to device type, surgical procedure, outcome definitions, and measures of thermal injury. An updated synthesis is therefore warranted to clarify the current evidence and identify areas where clinically meaningful differences, safety concerns, and evidence gaps remain.

This review synthesizes the current evidence on the mechanical characteristics and clinical performance of surgical energy devices, with particular emphasis on ultrasonic, advanced bipolar, hybrid, and emerging energy technologies. It examines comparative outcomes across commonly performed general surgical procedures, including laparoscopic cholecystectomy, colorectal resection, thyroidectomy, mastectomy, hernia repair, and bariatric surgery, while also considering perioperative safety, thermal tissue effects, and clinically reported energy-related injuries. Finally, emerging developments, including artificial intelligence (AI)-assisted energy delivery and its potential role in reducing inadvertent injury to adjacent tissues, are discussed.

## Review

Methodology

The literature review was conducted after retrieving papers from peer-reviewed databases like PubMed using the search string given in the Appendices. The search was conducted in June 2026 using the following search criteria: Publication date: 2010/01/01-2026/06/01; Species: humans or animal studies when specifically noted in the HIFU/histotripsy sections. Studies published before 2020 were incorporated when they served as the foundation for contemporary background information (e.g., studies characterizing the device's physics). Studies were prioritized from January 2010 to June 2026 in the context of public health information and communication technology (ICT). Studies focused on adult patients undergoing general surgical procedures (open or laparoscopic/robotic) and using ultrasonic or acoustic energy devices such as Harmonic® systems (Ethicon, Johnson & Johnson), Sonicision systems (Covidien/Medtronic), Thunderbeat® systems (Olympus Corporation), HIFU, histotripsy, or equivalent acoustic/ultrasonic technologies were included. Operative time, blood loss, length of hospital stay, postoperative complications, thermal safety, and device performance were considered for narrative review reporting. The types of studies chosen were randomized controlled trials (RCTs), systematic reviews and meta-analyses, prospective or retrospective cohort studies, ex vivo comparative studies, and narrative reviews. However, studies were excluded if they reported only physiotherapy or diagnostic ultrasound without surgical dissection, were of non-general surgical specialties (e.g., cardiothoracic or ophthalmology) with no general surgical relevance, were case reports (n<5), or had no English full-text available.

Synthesis of evidence

Mechanisms of Action: How Ultrasonic Devices Work

This biophysical understanding of what is needed for ultrasonic tissue dissection is significant for appreciating certain clinical benefits of any device. The current passes through tissues, and tissue resistance can also cause some tissue damage (burns) under the pad on the patient, as well as cardiac interference with monopolar and bipolar electrosurgery. This cannot be accomplished by mechanically transmitting ultrasonic signals (transmitters). The blade's longitudinal mechanical vibration is generated by high-frequency electrical oscillation (for example, the Harmonic ACE® ultrasonic surgical system operates at approximately 55.5 kHz, whereas the ultrasonic component of the Thunderbeat® system operates at approximately 23.5 kHz), which is converted into mechanical vibration by the device handpiece's piezoelectric transducer [10,11].

The inflammatory response in the histologically examined areas of ultrasonic incisions is much less than that of electrosurgical incisions. Molecular-level evaluation revealed that the Harmonic® blade incisions had a lower proportion of genes associated with the hemostasis response, a lower number of genes associated with the inflammation/immune response, and more genes associated with wound healing/angiogenesis three days after the incisions were made, compared to incisions made with energized devices [10,12,13]. From this study's results, a molecular-level explanation for the clinically observed benefits of reduced pain and reduced drainage fluid after the ultrasonic dissection method can be drawn (Table 1).

**Table 1:** Biophysical characteristics of common surgical energy modalities HIFU, high-intensity focused ultrasound; N/A, not applicable; Max, maximum. Table credits: Created by the authors using [10-13]

Parameter	Monopolar Electrosurgery	Bipolar Electrosurgery	Ultrasonic Devices	HIFU
Energy type	Electrical (resistive heat)	Electrical (resistive heat)	Mechanical vibration	Focused acoustic energy
Operating frequency	300-500 kHz	300-500 kHz	23,000-55,500 Hz	0.5-7 MHz
Working temperature	300-400°C	100-200°C	80-150°C	Varies (thermal/cavitation)
Lateral thermal spread	Up to 10 mm	5-7 mm	1-3 mm	Focal, contained
Electrical current in patient	Yes	Yes	No	No
Max vessel sealing diameter	Variable	5 mm	5-7 mm	N/A (ablation)
Smoke/plume generation	High	Moderate	Minimal	None (transcutaneous)

Current Clinical Evidence

Comparing surgical effectiveness can help evaluate the skills acquired during surgery training. There has been a significant increase in clinical evidence supporting the use of ultrasonic devices in general surgery over the last seven years (2020-2026). As of 2023, an extensive umbrella review of studies evaluating the use of the Harmonic® scalpel over the last three decades across various surgical fields found that the findings have been consistent regarding operating time, blood loss, and postoperative complications [14]. 

A systematic review and network meta-analysis (NMA) in Techniques in Coloproctology directly compared LigaSure (Covidien/Medtronic), Harmonic®, Thunderbeat®, and conventional electrosurgery for general surgical procedures in 2022. This NMA demonstrated that LigaSure reduced operative time by a mean of 42.8 minutes (95% CI: -53.9 to -31.5 min), Harmonic® by 28.3 minutes (95% CI: -33.6 to -22.6 min), and Thunderbeat® by 26.1 minutes (95% CI: -46.0 to -6.0 min) compared to conventional techniques [15]. Compared with conventional hemostatic techniques, both LigaSure and Harmonic® were associated with significantly lower operative blood loss, with mean differences of -24.1 mL (95% CI, -46.54 to -1.58) and -24.6 mL (95% CI, -42.4 to -6.7), respectively. Hospital stay was also significantly shorter with LigaSure and Harmonic®, with mean differences of -1.3 days (95% CI, -2.2 to -0.4) and -0.5 days (95% CI, -1.0 to -0.1), respectively. No significant differences were identified between the energy devices for the remaining individual outcomes assessed [15]. While the specific attributes of these devices are impressive, the NMA concluded that "no device is superior overall," and all three energy platforms exhibited a "similar perioperative safety profile" [15]. 

Cholecystectomy and abdominal procedures: One of the first surgical procedures studied in which ultrasound dissection was found to be beneficial was laparoscopic cholecystectomy, for which there was generally a correlation between the use of ultrasound dissection instruments and reductions in operating time and bleeding complications. Recent bariatric surgery evidence bears this out, as an 8,157-patient retrospective study (5,143 using Thunderbeat® and 3,014 using Maryland LigaSure) of the laparoscopic sleeve gastrectomy showed that there was still no statistically significant difference in the overall postoperative bleeding rates (0.35% vs. 0.63%, respectively; P=0.069), confirming the overall safety of both platforms for high-volume bariatric surgery [16].

Mastectomy and breast surgery: A prospective comparative study of patients undergoing modified radical mastectomy (MRM) for breast carcinoma showed faster operating room time and reduced drain output in patients using the Harmonic® scalpel as compared to those using electrocautery [17]. In addition, a meta-analysis of the various types of flap procedures carried out in 2022 found that the use of the Harmonic® ultrasonic surgical system scalpel reduced the mean harvest time of the flap by 26.29 minutes (95% CI: -39.38, -13.2 min) and the mean drain volume by 58.76 mL (95% CI: -105.27, -12.25 mL) compared to conventional electrocautery [18]. There was no difference in bleeding volume, infection rate, flap necrosis, or wound dehiscence, indicating that the two were equally safe and efficient [18].

Colorectal surgery: In colorectal resection, evidence suggests that ultrasonic devices, particularly when combined with bipolar energy, as in the Thunderbeat®, provide safe and effective control of mesenteric vessels. A comparative study evaluating Thunderbeat® versus LigaSure and Harmonic® in laparoscopic colorectal resection found no significant differences in operative time (240 vs 246 minutes, P=0.779), intraoperative complications, conversion to open surgery, postoperative morbidity, mortality, or length of hospital stay, reinforcing the clinical equivalence of modern energy platforms in colonic procedures [19]. Notably, mean blood loss appeared less favorable with ultrasonic instrumentation, particularly in colorectal procedures, compared with other general surgical categories, suggesting procedure-specific variation in device effectiveness (Table 2).

**Table 2:** Key clinical outcomes by surgical procedure (2020-2026 evidence summary) MRM, modified radical mastectomy; NMA, network meta-analysis; LOS, length of stay. Manufacturer: Harmonic® (Ethicon, Johnson & Johnson); LigaSure (Covidien/Medtronic); Thunderbeat® (Olympus Corporation)

Procedure	Device Studied	Key Outcome	Finding	Reference
Flap harvesting (MRM)	Harmonic® vs electrocautery	Harvest time, drain volume	↓ 26.3 min, ↓ 58.76 mL drain	[18]
General surgery (NMA)	LigaSure, Harmonic®, Thunderbeat®	Operation time, blood loss, LOS	All superior to conventional	[15]
Sleeve gastrectomy	Thunderbeat® vs LigaSure	Postoperative bleeding rate	Equivalent (0.35% vs 0.63%)	[16]
Colorectal resection	Thunderbeat® vs LigaSure and Harmonic®	Operation time, morbidity, mortality	Equivalent across all metrics	[19]
Modified radical mastectomy	Harmonic® vs electrocautery	Operative time, seroma	↓ time, ↓ seroma with Harmonic®	[17]

Safety Profile and Complications

Thermal effects and lateral spread: Despite their superior thermal spread compared with electrosurgery, ultrasonic devices are not free of thermal risk. All ultrasonically activated devices generate blade temperatures exceeding 100°C during activation, with maximum cutting temperatures varying significantly by device: Harmonic ACE® reaches 191.1°C; Sonicision 227.1°C; and SonoSurg® 184.8°C (P<0.001) [20]. Cooling times to reach a safer 60°C range from 27.4 to 38.7 seconds depending on device, meaning retained thermal energy poses a significant risk of inadvertent tissue injury if the active blade is not carefully controlled between activations, a finding with direct implications for surgical training and technique [20,21].

An ex vivo study evaluating the direct and indirect thermal effects of multiple surgical energy devices found that for all devices tested, temperatures exceeding 46°C (the cellular injury threshold) and 60°C (the nerve injury threshold) were usually not reached more than 5 mm from the activation site [22]. This finding supports the concept that maintaining a minimum 5 mm clearance from critical structures during activation may constitute a safe operating principle for all energy devices currently in clinical use. Steam spread was also observed to increase with wider jaw grasping ranges, representing an additional source of off-target thermal injury that surgeons should account for during deep pelvic or retroperitoneal dissection [22].

Device-specific adverse events: A retrospective analysis of the U.S. Food and Drug Administration (FDA) Manufacturer and User Facility Device Experience (MAUDE) database covering adverse events involving Harmonic® and LigaSure between 2013 and 2023 revealed important safety distinctions [23]. Harmonic® devices demonstrated significantly higher rates of device overheating (6.9% vs 0.6%, P=0.003) and spontaneous self-activation (4.4% vs 0%, P=0.032) than LigaSure. In contrast, LigaSure was associated with significantly more intraoperative bleeding events than Harmonic® (8.2% vs 0.0%, P=0.001) [23]. These complementary risk profiles underscore the importance of device familiarity and surgeon vigilance, particularly with respect to temperature monitoring and activation protocols [23].

High settings and prolonged ultrasonic application (>10 seconds) substantially increase the risk of lateral thermal injury, particularly near delicate structures. Cooling times, the duration for blades to return to safe temperatures, differ significantly between devices (ACE®: 35.7 s; Sonicision: 38.7 s; SonoSurg®: 27.4 s) [21]. Understanding device-specific cooling profiles is a critical but often underemphasized component of safe energy device use (Table 3).

**Table 3:** Safety and thermal profiles of key ultrasonic devices N/A, not applicable; Max, maximum. Table credits: Created by the authors using [20-23]

Device	Manufacturer	Max Cutting Temperature (°C)	Active Blade Cooling Time (s)	Key Safety Concern
Harmonic ACE®	Ethicon, Johnson & Johnson	191.1	35.7	Device overheating; self-activation
Sonicision	Covidien/Medtronic	227.1	38.7	Highest max temperature among class
SonoSurg®	Olympus Corporation	184.8	27.4	Rapid cooling advantage
Thunderbeat®	Olympus Corporation	Dual-mode (ultrasonic + bipolar)	Variable	Integrated bipolar mitigates some risk
LigaSure (comparator)	Covidien/Medtronic	N/A (bipolar)	N/A	Intraoperative bleeding risk; not ultrasonic

HIFU in Surgical Oncology

In addition to traditional ultrasound dissectors, ultrasound energy can be focused to much higher intensity to create HIFU, a new and emerging ultrasound surgical application appropriate for oncology surgery. Therefore, with focused ultrasound (HIFU), it is possible to generate focal coagulative thermal necrosis and tissue destruction within a very localized volume without making any cuts in the skin. HIFU is not limited by the need to operate in the narrow space in the body required by laparoscopic or ultrasonic dissectors in surgery; it is a non-invasive surgical method and can be used to treat soft-tissue sarcomas and hepatocellular, pancreatic, breast, and uterine fibroids [24].

More recently, a preliminary study evaluating HIFU ablation for soft tissue tumors in anatomically challenging locations adjacent to critical nerves, blood vessels, and joint areas reported successful treatment of all 11 enrolled patients, with a median non-perfusion volume ratio of 93.24% after HIFU [24]. These results support expanding the clinical role of HIFU as a viable alternative for surgically high-risk or recurrent soft tissue lesions [25].

Pain during sonication is a recognized adverse event of MR-guided HIFU, reported in roughly a quarter of patients and occasionally severe enough to cause premature termination of sonication [26]. In contrast, more serious events, including pathological fractures, third-degree skin burns, and neuropathy, remain rare. Critically, more than 60% of reported adverse events resolved on the same procedure day, supporting an overall favorable safety profile for carefully selected patients [26].

Device Comparison and Selection Framework

Selecting the optimal ultrasonic or acoustic energy device for a given procedure requires integrating procedure-specific factors, anatomical considerations, vessel diameter, and the desired balance between cutting speed and coagulation. No single device has demonstrated categorical superiority across all surgical domains, and the choice between Harmonic®, Thunderbeat®, LigaSure, and their alternatives should be guided by procedural context and institutional availability.

The following summarizes a practical selection framework based on current evidence:

Harmonic ACE®/FOCUS® platforms are best suited for precision dissection in thyroid, parathyroid, and head/neck surgery where minimizing thermal spread is paramount; these have the lowest intraoperative bleeding event rate among energy devices [27].

LigaSure advanced bipolar systems have superior operative time efficiency and are ranked first for speed across general surgical procedures in NMA; they are appropriate for colorectal, hernia, and bariatric surgery where large tissue volumes require rapid sealing [15].

Thunderbeat® advanced energy systems are optimal for procedures requiring both precise dissection and reliable vessel sealing ≤7 mm; integrating ultrasonic and bipolar energy reduces switching between instruments during laparoscopic colorectal and sleeve gastrectomy cases [28].

The Sonicision cordless ultrasonic dissection system is validated for laparoscopic and open procedures that prioritize device manoeuvrability and ergonomics, achieving a 97.6% surgeon satisfaction rate with no steam-related visual obstruction encountered in 95% of cases [29].

HIFU reserves non-invasive ablation of solid tumors for patients unsuitable for conventional surgery, with current indications including pain palliation, complete ablation of localized early-stage tumors, and debulking of surgically unresectable late-stage cancers; it requires dedicated imaging guidance (ultrasound or magnetic resonance imaging (MRI)) and multidisciplinary team planning [30].

Future Directions

AI-powered smart energy delivery systems: The most anticipated technological advancements in surgical energy platforms on the verge of becoming real game-changers include the integration of AI. Machine learning algorithms are now embedded in electrosurgical and ultrasonic generators and can help automatically adjust the energy level, provide real-time tissue characterization, and deliver real-time warnings during the surgical procedure if a key safety parameter (such as the amount of energy delivered) exceeds its safe limits. AI-powered energy tools can minimize the incidence of unintended thermal damage, enhance control over bleeding, and provide more consistent surgical performance across different skill levels and more uniform surgical results across surgeons [31].

Robotic-integrated ultrasonic platforms with wristed articulation: There has been a major gap in robotic surgery, specifically the lack of a fully integrated wristed ultrasonic energy instrument that offers the flexibility of articulated wrist motion, as well as ultrasonic cutting and coagulation features. The proposed integration of robotic surgery now puts the spotlight on the potential for AI-assisted surgical guidance: the da Vinci 5® robotic surgical system (Intuitive Surgical, Inc.), which was also FDA-approved in 2024, with additional software approved in 2025, is designed to be AI-ready and incorporates capabilities that enable AI-assisted surgical guidance, including force feedback, which could help with intelligent energy delivery [32].

Expanding HIFU applications and immunomodulatory potential: Besides these possibilities for successive tissue ablation, HIFU is also an emerging technology with potential applications beyond ablation, such as sono-immunotherapy, which involves slowing or stopping tumor growth, thereby triggering a local immune response [33]. This immunological component of HIFU is a new area of opportunity, where surgery, oncology, acoustic physics, and cancer immunology converge.

Histotripsy (non-thermal acoustic tissue destruction): A developing HIFU instance is a non-thermal cavitation method called histotripsy, in which high-amplitude, short-duration ultrasound pulses that generate little or no heat are focused within the tissue and used to homogenize it [34]. Feasibility and safety studies have already been completed, and the clinical translation of the method for treating hepatocellular carcinoma (HCC) and other abdominal cancers is underway (Table 4). 

**Table 4:** Emerging technologies and their clinical potential in acoustic/ultrasonic surgery AI, artificial intelligence; MR, magnetic resonance; HIFU, high-intensity focused ultrasound; BBB, blood-brain barrier; MRI, magnetic resonance imaging. Table credits: Created by the authors using [31-34]

Technology	Current Status	Potential Application	Key Challenge
AI-guided adaptive energy delivery	Early commercial deployment	Automated hemostasis optimization	Regulatory validation; algorithm transparency
Robotic-integrated wristed ultrasonic	Preclinical/early commercial (2026)	Robotic colectomy, prostatectomy	Mechanical durability; cost
MR-guided HIFU	Established for fibroids/prostate; expanding	Soft tissue sarcoma, pancreatic tumors	Access to MRI suites; treatment duration
Histotripsy	Early clinical trials	Liver, kidney tumor ablation	Targeting precision; real-time feedback
Sono-immunotherapy (HIFU + immune)	Preclinical/pilot studies	Oncological debulking + immune priming	Optimal acoustic parameters undefined
Nano-enhanced HIFU (microbubbles)	Research phase	Targeted drug delivery across BBB	Safety of microbubble formulations

Limitations of Current Evidence and Research Gaps

Although there is a large body of literature on ultrasonic energy devices, significant gaps remain. First, few comparative studies include both immediate operative results (blood loss, operative time, and complications) and follow-up data on long-term oncological outcomes, recurrence, or quality of life. Second, the literature up to 2026 shows almost no head-to-head RCTs of the latest generation of AI-integrated energy devices versus conventional ultrasonic energy devices. Third, the rates of thermal injury events and adverse event data available by device type have been inconsistent across published reports, and there has been no standard definition for reporting thermal injury adverse events, making it difficult to conduct pooled analyses and to establish clear thresholds. Additionally, the majority of the large-scale comparative studies have been performed in academic, high-volume settings in the high-income world, and the applicability of the results to other surgical community settings, where training and technique may differ and where the volume of surgical procedures may be lower, is unclear, as device performance could be different.

Future research should prioritize prospective and multicenter evaluation of energy-related tissue injuries using standardized definitions and reporting criteria, alongside systematic documentation of uncommon injuries through case reports and case series. Studies should also assess delayed complications and the potential role of AI-assisted energy delivery systems in reducing inadvertent injury to adjacent tissues.

## Conclusions

Acoustic and ultrasonic energy devices are well established as instruments in general surgery, and a growing body of evidence shows benefits compared to traditional electrosurgery in select aspects of intraoperative performance, such as operative time, blood loss, and inflammatory response at the tissue level. Although no single surgical energy device has been established as universally superior, NMA-level evidence suggests that specific devices, including Harmonic®, LigaSure, and Thunderbeat®, may offer advantages for selected surgical outcomes and procedures. These findings should be interpreted in the context of the specific comparator, surgical procedure, and outcome assessed, rather than as evidence of overall superiority across all areas of surgery. Modern energy platforms generally demonstrate comparable perioperative safety profiles, although differences in blade and device heating, thermal retention, and cooling time after activation remain clinically relevant considerations. These parameters should therefore be incorporated into education and training on the safe use of surgical energy devices.

## Appendices

**Table 5:** Search string

Database	String
PubMed	("ultrasonic surgical device" OR "ultrasonic scalpel" OR "harmonic scalpel" OR "acoustic energy surgery" OR "laparoscopic ultrasonic dissection" OR "Thunderbeat" OR "LigaSure" OR "soft tissue dissection" OR "HIFU surgery" OR "energy-based devices" OR "vessel sealing") AND ("general surgery" OR "laparoscopic surgery" OR "minimally invasive surgery" OR "cholecystectomy" OR "colorectal resection" OR "thyroidectomy" OR "mastectomy" OR "bariatric surgery") AND ("complications" OR "hemostasis" OR "operative outcomes" OR "clinical trial" OR "systematic review" OR "meta-analysis")
